# Clinical and epidemiological features of the 2014 large-scale dengue outbreak in Guangzhou city, China

**DOI:** 10.1186/s12879-016-1379-4

**Published:** 2016-03-01

**Authors:** Yong Ping Lin, Yasha Luo, Yuan Chen, Mart Matthias Lamers, Qiang Zhou, Xiao Han Yang, Sumana Sanyal, Chris Ka Pun Mok, Zhong Min Liu

**Affiliations:** Department of Laboratory Medicine, The First Affiliated Hospital of Guangzhou Medical University, Guangdong, China; Research Centre of Translational Medicine, The First Affiliated Hospital of Guangzhou Medical University, Guangdong, China; Department of Laboratory Medicine, The Second Affiliated Hospital of Guangzhou University of Chinese Medicine, Guangdong, China; HKU-Pasteur Research Pole, School of Public Health, HKU Li Ka Shing Faculty of Medicine, The University of Hong Kong, Hong Kong, China; Department of Laboratory Medicine, Guangdong Women and Children Hospital, Guangzhou, China

**Keywords:** Dengue, China, Clinical, Outbreak, Endemic

## Abstract

**Background:**

Dengue virus is transmitted by mosquito around the tropical and sub-tropical regions. There was a large-scale dengue epidemic in Guangdong province, China during 2014 and around fifty thousands dengue fever cases, including six deaths, have been reported. In this study, we aimed to understand the clinical characteristics of hospitalized patients with laboratory-confirmed dengue virus (DENV) infection and determined the origin of the virus from the outbreak.

**Methods:**

We have summarized the data from 138 hospitalized patients who were laboratory confirmed for dengue infection in Guangzhou city. Patients were classified as either non-severe dengue fever or severe dengue fever according to the guidelines from the WHO. Viral serotypes were determined by real time RT-PCR. Genetic sequences of the envelope and non-structural genes were amplified and analyzed from the serum samples of eleven patients.

**Results:**

Co-circulation of dengue serotype 1 and 2 were identified from the outbreak. Patients infected by serotype 1 or 2 showed similar clinical features. Patients with severe dengue fever showed prolonged hospitalization and significant impairment of organ functions. Four samples from serotype 1 and five samples from serotype 2 were closely related respectively and clustered with Guangzhou isolates from previous years. The remaining isolates of serotype 1 were related to viruses found in Malaysia, India, Bangladesh and Singapore.

**Conclusion:**

The phylogenetic grouping of Guangdong isolates suggests that dengue is no longer an imported disease in China. Analysis of the isolates obtained in this study together with the size of the outbreak are suggestive of endemic circulation in Guangdong province.

**Electronic supplementary material:**

The online version of this article (doi:10.1186/s12879-016-1379-4) contains supplementary material, which is available to authorized users.

## Background

Dengue virus is a mosquito-borne pathogen and mainly transmits around the tropical and sub-tropical regions. It belongs to the family of *Flaviviridae* and is an enveloped, positive sense single-stranded RNA virus with the size of around 11 kb. Infection of either one of the four serotypes causes mild to severe disease symptoms [1]. It is estimated that around 50 to 100 millions people were infected by the dengue viruses every year and over 100 countries are being affected [2, 3]. In mainland China, outbreaks of dengue fever mainly occurred in the southern area (i.e. Guangdong, Yunnan) [4, 5]. Dengue infection in China was first reported in Guangdong province in 1978. From 1978 to 1989, Guangdong province was suffered from several times of serious dengue outbreaks [6]. Up-to-date, all four serotypes have been identified in Guangdong province but DENV-1 is still the main serotype circulating in the area. It has been suggested that the outbreaks occurred in China were mainly initiated by the import cases from Southeast Asian countries [7]. Although cases of dengue infection have been identified in every year, China has not experienced any large DENV outbreak (more than 10,000 cases) since 1990 [8]. However, from July to November of 2014, there was an unusual large-scale dengue outbreak in Guangdong province. Around fifty thousands of dengue fever cases have been reported, resulting in six deaths. The objective of the study was to describe the clinical characteristics of hospitalized patients with laboratory-confirmed dengue infection and determine the origin of the virus from the outbreak.

## Methods

### Patients and sample collection

Hospitalized patients diagnosed as laboratory-confirmed DENV infection at the First Affiliated Hospital of Guangzhou Medical University were included in this study. Dengue fever cases were confirmed either by real time reverse transcription-polymerase chain reaction (DAAN, China), dengue NS1 antigen ELISA detection (WANTAI, China) or the IgM/IgG capture ELISA kits (PanBio) from the serum samples of the suspected cases. At the time of enrolment and hospitalization, the subject’s clinical history, physical examination, hematological, biochemical and microbiological investigations were recorded. Presumed day of onset was defined from the first day of presenting fever. Severe dengue cases were classified as those who determined as laboratory-confirmed DENV infection and showed severe bleeding, severe plasma leakage or severe organ involvement according to the guidelines provided by the WHO [2]. We determined further investigation of severe plasma leakage by several criteria: 1) High or progressively rising haematocrit; 2) Pleural effusion or ascites; 3) circulatory compromise or shock (tachycardia, cold and clammy extremities, capillary refill time greater than three seconds, weak or undetectable pulse, narrow pulse pressure or, in late shock, unrecordable blood pressure. This study was approved by the ethics committee of the First Affiliated Hospital of Guangzhou Medical University (2015–8) which waived the need for written consent since virologic testing was a routine diagnostic procedure and patient information saved at the study database was delinked from individual patient identifiers.

### DENV serotyping

Dengue virus serotyping was determined by real time PCR kit with in-house designed primers: dengue 1: Forward : 5’-TCAATATGCTGAAACGCGCGAGAAACCG-3’, Reverse: 5’-CGTCTCAGTGATCCGGGGG-3’; dengue 2: Forward: 5’-TCAATATGCTGAAACGCGCGAGAAACCG-3’, Reverse: 5’-CGCCACAAGGGCCATGAACAG-3’ which were targeted to the envelope gene. Reaction conditions were performed by the Bio-Rad CFX96 real-time PCR system.

### Genomic analysis

The full genomes of E and NS1 genes of the serotype 1 and 2 viruses were amplified by RT-PCR and the sequences of the PCR products were identified by sanger sequencing method. The sequences have been uploaded to Genebank (accession numbers: KT751340-KT751363).

### Phylogenetic analysis

Multiple sequence alignment was performed using ClustalW software. Phylogenetic trees were drawn using the Maximum likelihood method by the TN93 model with gamma-distribution of among-site in MEGA 6.06 (www.megasoftware.net). Bootstrap analyses with 1,000 re-samplings were used to determine confidence values for groupings within the phylogenetic trees (Additional files 1, 2, 3 and 4).

### Statistical analysis

Descriptive statistics for the study population characteristics and laboratory findings were performed using SPSS Inc. (Chicago, IL, Version 17.0). Quantitative data are presented as the mean ± standards deviation (SD) and are compared by non-pair student *t* test. The categorical variables are reported as frequencies and percentages following comparison with chi-square test.

## Results

Total of 387 hospitalized patients who developed fever higher than 38 °C with unknown origin between the 20th of September and the 20th of November 2014 were suspected as dengue fever, of which 138 (35.7 %) were positive for dengue infection. 130 (94.2 %) of the patients were classified as non-severe dengue fever (NSDF) and 8 (5.8 %) were severe dengue fever (SDF) (4 DENV-1, 1 DENV-2, 3 Unidentified). Two patients with SDF died during hospitalization. Among the 138 cases, 107 (77.5 %) and 17 (12.3 %) were infected by serotype 1 and 2 respectively while 14 were unidentified due to the negative detection of vRNA from the serum samples.

The mean age of the patients with SDF was 62.9 ± 27.7 years which was higher than the age of patients with NSDF (DENV1: 56.1 ± 22.1; DENV2: 57.2 ± 23.6). However, there was no statistical difference between the two groups (p > 0.05). Moreover, the mean age of the hospitalized patients with dengue infection was higher than that of non-hospitalized patients (mean age: 40.2 ± 19.5 years, p < 0.05). The period of hospitalization of patients with SDF was significantly longer than that of patients with NSDF (SDF:18.6 ± 9.5 vs NSDF: DENV1 8.8 ± 4.0/DENV2 8.9 ± 3.3 days, p < 0.05). Patients with dengue infection commonly showed typical clinical symptoms including fever, nausea, vomiting, diarrhea, abdominal pain, rash and aches/pains (Table 1). Among the severe cases, 2 (25 %) developed shock while 6 (75 %) showed impaired consciousness (Table 1).

**Table 1:** The clinical features and laboratory tests from the patients with dengue infection

	Non-severe dengue	Severe dengue	*P*
Total numbers	*N* = 130	*N* = 8*	N.D.
Age (years)	55.7(51.8 ~ 59.5)	62.9(35.2 ~ 90.6)	N.S.
Sex(Male) [N(%)]	59(45.4 %)	6(75 %)	N.S.
Number of deaths	0	2(25 %)	N.D.
Days of hospitalization	8.8(8.1 ~ 9.5)	18.6(10.7 ~ 26.6)	<0.001
Clinical symptoms	*N* = 122	*N* = 8	
Fever [N(%)]	122(100 %)	8(100 %)	N.S.
Temperature (°C)	39.1(39.0 ~ 39.2)	39.5(39.1 ~ 40.0)	N.S.
Nausea [N(%)]	32(26.2 %)	2(25 %)	N.S.
Vomiting [N(%)]	21(17.2 %)	2(25 %)	N.S.
Diarrhea [N(%)]	26(21.3 %)	2(25 %)	N.S.
Abdominal pain [N(%)]	23(18.9)	2(25 %)	N.S.
Rash [N(%)]	42(34.4 %)	3(37.5 %)	N.S.
Aches/pains [N(%)]	67(54.9 %)	4(50 %)	N.S.
Shock [N(%)]	0	3(37.5 %)	<0.001
Impaired consciousness [N(%)]	0	6(75 %)	<0.001
Laboratory tests	*N* = 120	*N* = 7	
WBC(4–10 × 109) ※	2.71(2.54 ~ 2.87)	2.47(1.70 ~ 3.24)	N.S.
Leukopenia [N(%)]	108(90 %)	8(100 %)	N.S.
PLT(100–300 × 109) ※	71(65 ~ 77)	30(4 ~ 55)	0.003
Thrombocytopenia [N(%)]	102(85 %)	7(100 %)	N.S.
Hct rising [N(%)]	6(5.0 %)	2(28.6 %)	<0.001
ALT(5-40U/L) ※	47.2(39.5 ~ 55.0)	138.3(0–290.5)	<0.001
AST(5-40U/L) ※	65.8(56.2 ~ 75.5)	179.5(70.8 ~ 288.2)	<0.001
D-Dimer(68–494 ng/ml) ※	1769(1366 ~ 2172)	5114(1449 ~ 8779)	<0.001
LDH(109-255U/L) ※	279(262 ~ 297)	2743(0 ~ 8430)	<0.001
CK(10-190U/L) ※	273(210 ~ 336)	1970(0 ~ 5504)	<0.001
Cr(33-133umol/L) ※	76.9(72.4 ~ 81.3)	276.4(0 ~ 655.1)	<0.001
Bun(2.9–7.2 mmol/L) ※	4.3(3.8 ~ 4.7)	10.0(3.7 ~ 16.4)	<0.001
Urine Protein(<0.3 g/L) ※	0.4(0.3 ~ 0.5)	1.0(0.3 ~ 1.8)	0.007

*: Four, Den1; One, Den2; Three, Undefined; ^※^: Normal range; N.S.: No significant; N.D.: Not detected

The laboratory diagnosis showed that the levels of alanine aminotransferase (ALT) and aspartate aminotransferase (AST), lactate dehydrogenase (LDH), D-Dimer, creatine kinase (CK) and urine protein were higher than the normal range in patients either with NSDF or SDF (Table 1). All of the clinical parameters listed above, together with levels of the creatinine (Cr) and blood urea nitrogen (Bun), were 2–3 folds higher in SDF patients compared to NSDF patients (Cr and Bun levels are normal in NSDF group) (p < 0.05). Majority of the patients, in both the NSDF and SDF group, showed leukopenia and thrombocytopenia, while the platelet count of SDF patients was significantly lower than that of DF patients (29.5 ± 29.8 vs DENV-1 69.9 ± 35.8/DENV-2 65.9 ± 31.2, p < 0.05).

**Fig. 1:**
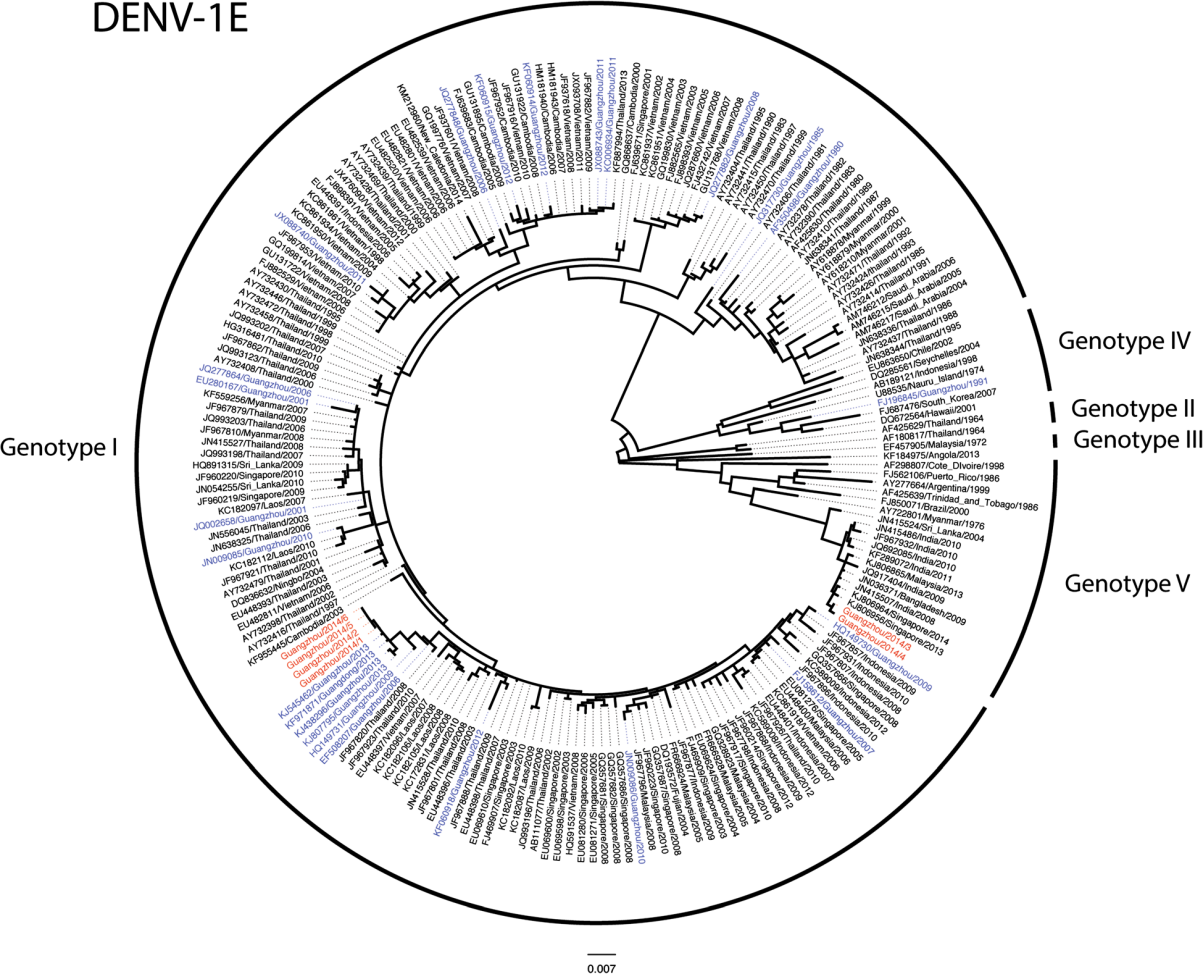
Phylogenetic tree of the complete E gene of DENV-1 isolates. Maximum likelihood trees were constructed by the TN93 + G model in MEGA 6.06 (http://www.megasoftware.net). Bootstrap values were calculated on 1,000 replicates. The DENV-1 isolates obtained in this study are in red and other isolates from Guangdong province are in blue. Scale bars indicate nucleotide substitutions per site

The envelope (E) and non-structural 1 (NS1) genes from the serum samples of 11 patients (6 from serotype 1 and 5 from serotype 2) were amplified and sequenced. Phylogenetic analysis showed that the DENV-1 viruses belonged to two separate groups. Four samples from serotype 1 belonged to genotype 1 and were closely related to viruses from Guangzhou, detected between 2006 and 2013 (99.3–99.9 % nt identity in E) (Figs. 1 and 3a). The closest relatives of these isolates were several strains isolated in Guangzhou in 2013 (accession numbers KJ545462, KJ807795, KJ438296) with E gene nucleotide identities of 99.7–99.9 %. The remaining two DENV-1 isolates belonged to genotype 5 and clustered with isolates from Malaysia (KJ806865), India (JN415507), Bangladesh (JN036371) and Singapore (KJ806964), with nucleotide identities between 99.3 and 99.6 % nt in E.

**Fig. 2:**
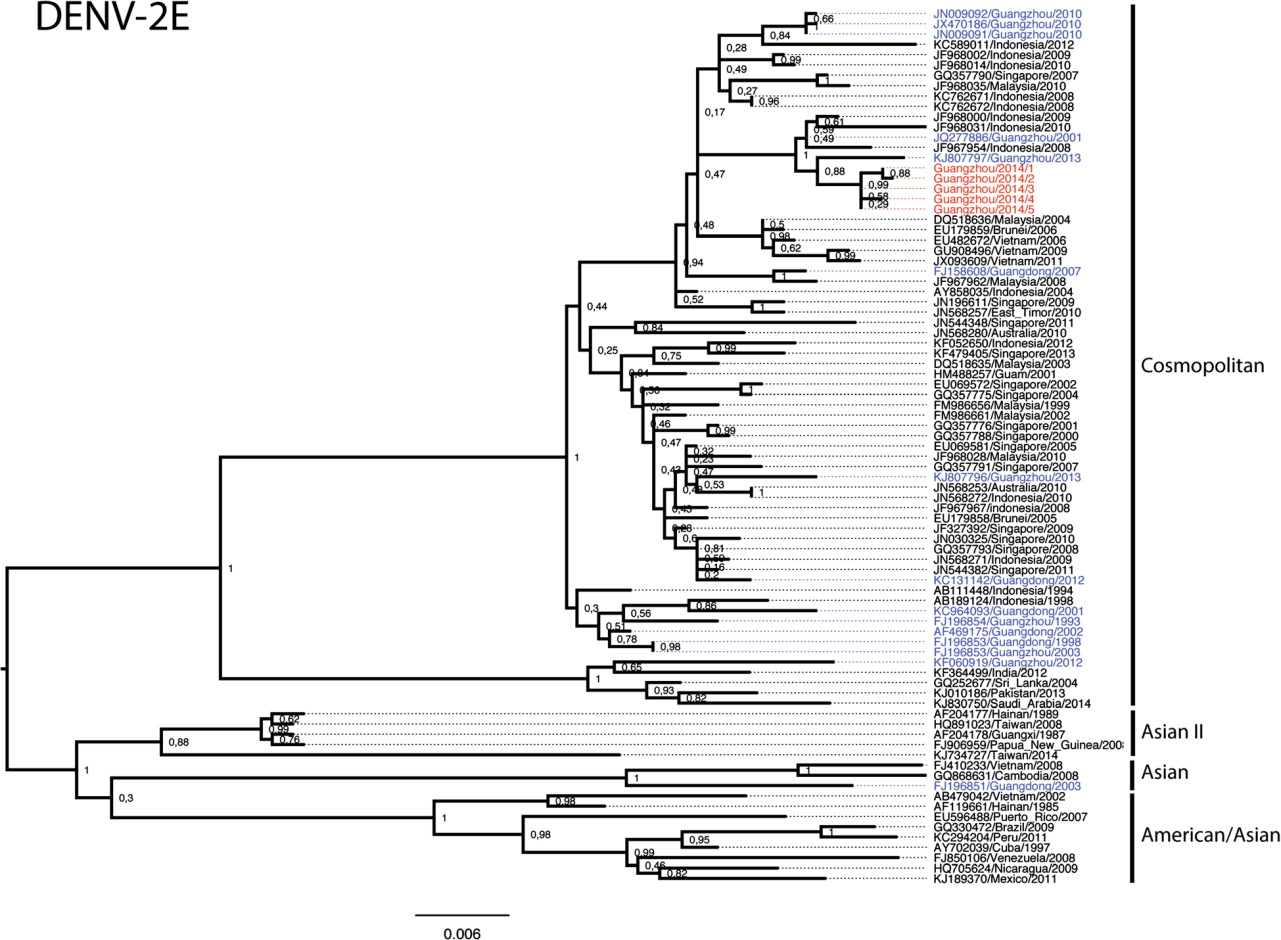
Phylogenetic tree of the complete E gene of DENV-2 isolates. Maximum likelihood trees were constructed by the TN93 + G model in MEGA 6.06 (http://www.megasoftware.net). Bootstrap values were calculated on 1,000 replicates. The DENV-2 isolates obtained in this study are in red and other isolates from Guangdong province are in blue. Scale bars indicate nucleotide substitutions per site

Phylogenetic analysis of the E gene indicated that the DENV-2 isolates belonged to the Cosmopolitan genotype (Fig. 2). All five isolates clustered monophyletically with DENV-2 sequences from virus strains circulating in Guangzhou in 2001 and 2013 (JQ277886, KJ807797; 98.9–99.5 % nt identity in E) and with Indonesian strains between 2008 and 2010 (Fig. 2) (JF967954, JF968000, JF968031; 98.5–99.3 % nt identity in E).

**Fig. 3:**
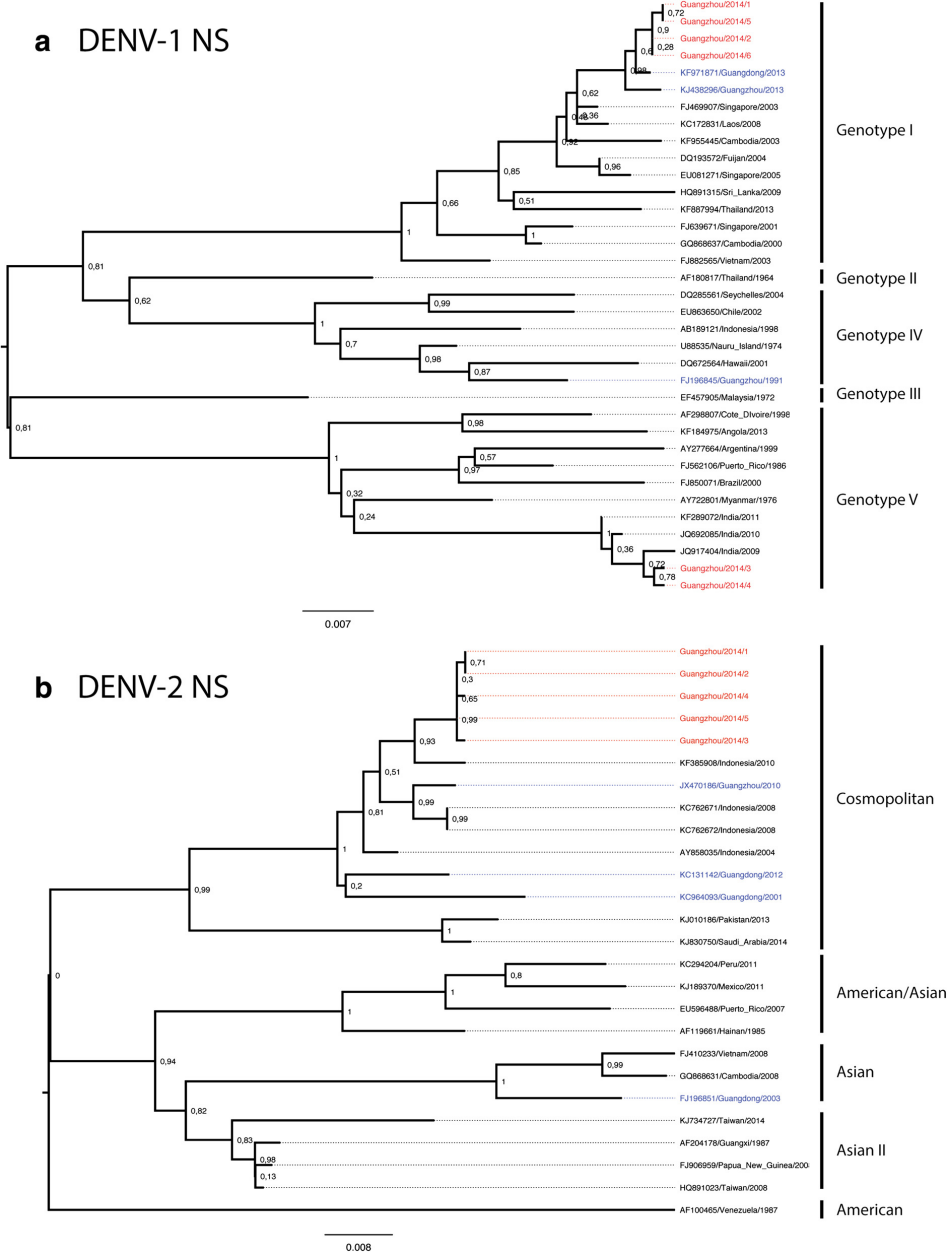
Phylogenetic tree of the complete NS gene of DENV-1 (**a**) and DENV-2 (**b**) isolates. Maximum likelihood trees were constructed by the TN93 + G model in MEGA 6.06 (http://www.megasoftware.net). Bootstrap values were calculated on 1,000 replicates. The DENV-1 and DENV-2 isolates obtained in this study are in red and other isolates from Guangdong province are in blue. Scale bars indicate nucleotide substitutions per site

Phylogenetic analyses of the NS genes of the DENV-1 and DENV-2 isolates supported the clustering patterns of the E genes although for DENV-2 there is no sequences available to show the persistence of the strains in consecutive years in Guangdong (Fig. 3).

## Discussion

The dengue outbreak occurred in Guangdong province during 2014 is largest epidemic in China since 1990. Although Guangdong province has the highest rate of dengue virus infection among China and the four serotypes can all be found, the scales of the outbreaks were relatively small and occurred in limited areas throughout the past twenty years [9]. In GuangZhou city, DENV-1 was predominant over the past two decades but other serotypes can be identified in some years. Molecular epidemiological study showed that the DENV-1 isolated in GuangZhou belongs to genotype I, IV and V and the recent isolates are all belongs to I and V while almost all the DENV-2 isolated in GuangZhou belongs to Cosmopolitan genotype [10].

The factors that contribute to this epidemic in 2014 are still not very clear. Although *A. aegypti* is known as the most important vector of dengue viruses, *A. albopictus* is predominant in Southern China and found as the major species to carry the dengue virus [9]. Changing of the activity of *A. albopictus* by urbanization and global climate were suggested to associate with the dengue transmission [11]. Recent study using mathematic modeling as analysis approach showed that the mosquito density and temperatures influence the development of this dengue epidemic in Guangzhou city [12]. However, comprehensive studies from epidemiology, virology and surveillance are required to further understand the cause of the outbreak.

Majority of the patients admitted to our hospital showed typical but mild dengue fever. Even in the limited severe cases, development of severe haemorrhagic manifestation, plasma leakage or shock was rarely seen. Our results showed that there was no significant difference on clinical features or laboratory tests between the patients infected with serotype 1 or 2. Several studies suggested that the risk of causing severe dengue infection is lower in serotype 1 compared to other three serotypes [13–16]. While there were only 8 (5.8 %) patients who developed severe dengue fever, most of them were older than the group with mild disease [Mean age: 55.7 vs 62.9 (years old)]. We believe that the reason of the disease progression from the SDF group may mainly due to the older in age instead of the serotypes. Interestingly, among 6 out of 8 (75 %) patients with severe dengue fever showed impairment of consciousness during hospitalization. This is a common presentation of DHF/DSS with prolong and profound shock and organ(s) failure, especially liver failure. Hepatic encephalopathy is likely to be the causes of this impaired consciousness. This was confirmed by higher degree of AST elevation and BUN, Creatinine values of SDF compare to NSDF. Other causes may be electrolyte (hyponatremia) or metabolic (hypoglycemia) disturbance. Some studies suggested that this clinical sign may associate to the disease progression and mortality during dengue infection [17, 18]. However, the diagnosis of impaired consciousness to predict the progression of dengue infection is needed to further investigated.

While several outbreaks of DENV in China were caused by imported cases from Southeast Asia, the size of this dengue epidemic in Guangzhou may indicate the establishment of endemic DENV transmission [19–21]. Our results support this notion since the majority of the serotype 1 isolates and all serotype 2 isolates we obtained clustered in the same clade and were most related to isolates found in Guangdong in the past successive years. Two serotype 1 isolates were not detected previously in Guangdong and these clustered with isolated found in India, Bangladesh, Singapore and Malaysia. This indicates that part of the epidemic may have been caused by the import of viruses from Southeast Asia and subsequent local transmission. In a recent report, the authors concluded that the dengue is overall still an imported disease in China [7]. The authors came to this conclusion due to the lack of Chinese isolates that clustered together in their phylogenetic analysis. However, in this analysis the authors did not include any isolates from the major epidemic in Guangdong in 2014 and therefore they failed to identify the monophyletic clustering of the Guangdong DENV-1 and DENV-2 isolates with viruses from previous years.

## Conclusion

In conclusion, our results showed the general clinical features from the patients infected by the 2014 dengue outbreak in Guangdong, China. The phylogenetic grouping of Guangdong isolates suggests that dengue is no longer an imported disease in China. Analysis of the isolates obtained in this study together with the size of the outbreak are suggestive of endemic circulation in Guangdong province.

## Additional files

Additional file 1:
**Alignment file of DENV1 E gene.** (TXT 292 kb) Additional file 2:
**Alignment file of DENV2 E gene.** (TXT 121 kb) Additional file 3:
**Alignment file of DENV1 NS1 gene.** (TXT 33 kb) Additional file 4:
**Alignment file of DENV2 NS1 gene.** (TXT 26 kb)

## Abbreviations

DENV: dengue virus
NS1: non-structural protein 1
SD: standards deviation
E: envelope
NSDF: non-severe dengue fever
SDF: severe dengue fever
ALT: alanine aminotransferase
AST: aspartate aminotransferase
LDH: lactate dehydrogenase
CK: creatine kinase
Cr: creatinine
Bun: blood urea nitrogen
DHF: dengue hemorrhagic fever
DSS: dengue shock syndrome
